# Added Value of Histological Evaluation of Muscle Biopsies in Porcine Vascularized Composite Allografts

**DOI:** 10.3390/jcm13175167

**Published:** 2024-08-30

**Authors:** Kaj Brouwers, Shannen R. W. M. van Geel, Dominique van Midden, Anne Sophie Kruit, Benno Kusters, Stefan Hummelink, Dietmar J. O. Ulrich

**Affiliations:** 1 Department of Plastic and Reconstructive Surgery, Radboud University Medical Center, 6525 GA Nijmegen, The Netherlands; 2 Department of Pathology, Radboud University Medical Center, 6525 GA Nijmegen, The Netherlands

**Keywords:** vascularized composite allografts, machine perfusion, static cold storage, ischemia–reperfusion injury, Histology Injury Severity Score, hematoxylin and eosin, nicotinamide adenine dinucleotide, membrane attack complex

## Abstract

**Background**: Machine perfusion (MP) offers extended preservation of vascularized complex allografts (VCA), but the diagnostic value of histology using hematoxylin and eosin (H&E) in detecting ischemia–reperfusion injury (IRI) in muscle cells remains unclear. This study aims to document the application of the Histology Injury Severity Score (HISS) and to assess whether additional staining for nicotinamide adenine dinucleotide (NADH) and membrane attack complex (MAC) improves IRI detection in a porcine limb replantation model. **Methods**: The forelimbs of 16 Dutch Landrace pigs were amputated and preserved for 24 h using hypothermic MP (*n* = 8) with Histidine-Tryptophan-Ketoglutarate (HTK) or for 4 h with SCS (*n* = 8) before heterotopic replantation and 7 days of follow-up. Muscle damage was assessed via biochemical markers and light microscopy using H&E, NADH, and MAC at baseline, post-intervention, and post-operative day (POD) 1, 3, and 7 timepoints, using the HISS and a self-developed NADH and MAC score. **Results**: H&E effectively identified damaged muscle fibers and contributed to IRI assessment in porcine limbs (*p* < 0.05). The highest HISS was measured on POD 3 between MP (4.9) and SCS (3.5) (*p* = 0.029). NADH scores of both preservation groups varied over the 7-day follow-up and were statistically insignificant compared with baseline measurements (*p* > 0.05). MAC revealed no to minimal necrotic tissue across the different timepoints. **Conclusions**: This study documents the application of the HISS with H&E to detect IRI in muscle fibers. NADH and MAC showed no significant added diagnostic utility. The 24 h MP showed similar muscle alterations using the HISS compared to that of the 4 h SCS after a 7-day follow up.

## 1. Introduction

Vascularized composite allografts (VCA) involve transferring multiple tissue types such as skin, bone, and muscle [1]. In recent years, there has been a substantial increase in clinical interest in VCAs, particularly for patients whose injuries exceed the scope of routine plastic surgery, such as complex trauma to the hand or face [2]. VCAs are highly complex and technically challenging procedures to perform, further compounded by the scarcity of available donors. In addition to this, muscle tissue has a limited ischemia time, as it is more prone to ischemia–reperfusion injury (IRI) compared to other tissues [3]. The current gold standard for VCA preservation is static cold storage (SCS), during which the tissue is stored on ice at approximately 4 °C for a period of 4–6 h before transplantation. Exceeding this time frame will lead to irreversible damage to muscle and nerve tissue [4]. Hypothermic machine perfusion (MP) has shown promising results in extending tissue preservation to 12 h or even up to 24 h in experimental porcine [5,6,7,8,9,10] and human limb [11,12] models by providing a constant flow of preservation fluid with oxygen and nutrients and flushing out toxic agents [13].

In addition to clinical interpretation and biochemistry, histology serves as a commonly used method for determining muscle vitality and thereby, the assessment of IRI in VCAs. Previous research regarding experimental VCAs have shown how hematoxylin and eosin (H&E) can be used for the assessment of muscle damage using the Histology Injury Severity Score (HISS), as described by Müller et al. [10] and simplified by Kruit et al. [5]. The application of this simplified HISS has not yet been described in detail, making reproducibility challenging. This study aims to provide a practical appraisal of the HISS, reviewing its assessment and the translation of findings to clinical considerations. H&E is used to analyze the morphology of the muscle cell by staining the nuclei, the extracellular matrix, and the cytoplasm [14]. Although H&E staining serves its purpose, it does not reveal subtle deviations such as the myofibrillar architecture and intracellular mucins (glycoproteins) [15]. It can also be challenging to differentiate between different types of myocyte injury (e.g., myocyte necrosis). Thus, although identification of necrotic fibers via H&E is characteristic for IRI-induced muscle damage, other staining methods might be useful to detect muscle damage at an earlier stage. 

An alternative to H&E is nicotinamide adenine dinucleotide (NADH), which is an oxidative enzyme staining. NADH can show various structural abnormalities of muscle fibers, including the myofibrillar architecture and mitochondria, and is therefore valuable in diagnosing mitochondrial myopathies [16]. Additionally, NADH is used to evaluate sarcoplasmic structural details [16]. It utilizes enzymatic activity to release hydrogen from NADH, resulting in the production of a purple–blue formazan pigment that serves as a visual marker of the reaction site [17]. The intensity of the staining is proportional to the number of mitochondria and relates to the amount of cell damage [16]. NADH has been shown to be useful in animal studies (using rats) for assessing the viability of muscle fibers [18]. Another alternative is the use of membrane attack complex (MAC). MAC is a stain for the C5b-9 complement that accentuates necrotic muscle fibers, an early marker of necrosis leading to cell lysis [19]. 

The aim of this study is to comprehensively document the simplified HISS [5] and its application during machine perfusion of VCA, drawing upon our accumulated experience and expertise over time. This will be achieved by supplementing representative histological images alongside the scores. Finally, the NADH and MAC staining techniques will be evaluated to explore their potential added value in comparison to the commonly used H&E.

## 2. Materials and Methods

### 2.1. Study Design

Sixteen female Dutch Landrace pigs of 3–4 months old (35–60 kg body weight) were included in this study. The animals were housed under standard conditions, with access to food and water ad libitum. The experiments involving animals were performed with the approval of the Central Animal Laboratory and the Animal Ethics Board (project number 2016-0043 and 2021-0039) and in accordance with the local and national guidelines for animal care, following the ARRIVE guidelines. The animals were randomly assigned to one of the two study groups. The surgical procedures and the limb procurement in the axillar region were performed as previously described by our research group [5,20]. One group underwent limb preservation through SCS for 4 h (4 h SCS, *n* = 8) before heterotopic replantation using techniques previously described by our research group [5,20]. The limbs of the other group were attached to an open machine perfusion setup (24 h MP, *n* = 8), receiving a continuous supply of oxygenated, acellular heparinized (5000 IU) Histidine-Tryptophan-Ketoglutarate (HTK) solution for 24 h before heterotopic replantation. HTK was selected based on favorable outcomes observed in a previous study, wherein HTK showed superior histological results 7 days post-reperfusion in comparison to alternative solutions [20]. The HTK solution (Custodiol^®^; Koehler Chemi, Alsbach-Haenlien, Germany) was formulated with the additives polyethylene glycol (PEG) and L-glutamine, which benefit tissue preservation [21,22,23]. The pigs were monitored for 7 days following the surgery and were euthanized on day 7 using an overdose of phenobarbital. All the surgical procedures and measurements were conducted by the same researcher (K.B.).

### 2.2. Measurements

The weight of the limbs was measured after amputation, after the ex vivo preservation period, and on post-operative day (POD) 7, considering that both IRI and perfusion could impact the VCA weight increase [4]. The microvasculature of the limbs after MP was assessed using near-infrared (NIR) fluorescence angiography (PhotoDynamic Eye; Hamamatsu Photonics, Hamamatsu, Japan) after an intravenous injection of 5 mg of indocyanine-green (ICG, 5 mg/mL, Verdye, Diagnostic Green GmbH, Aschheim-Dornach Germany) prior to the intervention and on POD 3 and 7. During the ex vivo perfusion period, various parameters, including pressure, flow, temperature, and rate per minute (RPM), were monitored and registered on an hourly basis.

### 2.3. Specimen Procurement

At regular intervals of 2 h, samples of perfusion liquids were collected, both before and after they passed through the limbs, with the purpose of assessing ischemia-related muscle damage markers. Blood samples were collected before harvesting and on POD 1, 3, and 7. For the analysis of these samples, CG4+ and CG8+ iSTAT cartridges were used for the measurement of pH, pCO2, pO2, and lactate using an iSTAT blood analyzer (Abbott, Princeton, NJ, USA). The creatine kinase (CK) and potassium (K) levels in the perfusate samples were measured in a heparinized syringe using a 1256 Rapidlab Blood Gas Analyzer (Siemens Healthcare Diagnostics, Norwood, MA, USA). Muscle biopsy specimens were obtained every 6 h from the extensor and flexor muscles of the forelimb immediately prior to harvesting and subsequently at 6 h intervals throughout the MP period, following the intervention, and on POD 1, 3, and 7. 

### 2.4. Histological Analysis

Each muscle biopsy specimen was collected from the flexor and extensor muscle of the forelimb. Half of the biopsy specimens were fixated in formaldehyde and embedded in paraffin. The other half were frozen in liquid nitrogen and stored at −80 °C until further processing. The formalin-fixed, paraffin-embedded sections were cut into 3 µm thick sections and stained with standard H&E. All slides were evaluated using light microscopy for IRI, according to a modified version of the HISS [5] (range 0–12, Table 1) by a blinded pathologist. IRI was defined by the amount of interstitial edema, inflammation, variation in muscle fiber shape and size, and damaged muscle fibers. All features were scored and/or quantified on a whole slide basis using light microscopy (Leica DM 3000, Leica Microsystem Corp, Wetzlar, Germany) with ×20 magnification. The frozen sections were also cut into 3 µm thick cross sections in a cryostat and stained for NADH–tetrazolium reductase enzyme histochemical reaction and MAC. All slides were evaluated using light microscopy. The viability of muscle fiber was assessed by observing the myofibrillar architecture and intensity of the staining for NADH using a self-developed scoring system (range 0–3, Table 2), as a validated scoring system is lacking in the current literature. For MAC, each necrotic fiber was counted as a positive muscle fiber, and the muscle biopsies were scored based on the number of these positive fibers. Muscle cells with yellow granulation in both the cell membrane and endochylema, or exhibiting a yellow color in the vascular intima, were classified as positive for MAC using a self-developed scoring system (range 0–1, Table 3), as there is currently no validated scoring system in the existing literature.

### 2.5. Statistical Analysis 

The primary outcome of this study was the evaluation of muscle histology, with the collection of biochemical parameters as a secondary outcome. The ability of the different staining methods to identify muscle damage across different timepoints in both preservation groups was analyzed. In the case of normally distributed data, an ANOVA test was performed; in case of non-normally distributed data, an independent samples Mann–Whitney U test or an independent samples Kruskal–Wallis test was performed. In cases in which the data was not feasible for statistical comparison, only a descriptive analysis was performed. Continuous data are presented as the mean (standard deviation) and median. A 95% confidence interval was used, and a *p*-value < 0.05 was considered statistically significant. Data analyses were performed using SPSS 29.0 (IBM Corporation, New York, NY, USA) and Microsoft Excel 2016 (Microsoft Corp., Redmond, WA, USA).

## 3. Results

### 3.1. Baseline Characteristics

The baseline characteristics were similar across both intervention groups (Table 4) [24]. However, there was a difference in warm ischemia time (WIT) before storage between the groups. In the MP group, the WIT was 10.9 min longer compared to that of the SCS group (*p* = 0.005). This can be attributed to the process of cannulating, flushing, and attaching the limb to the perfusion machine. 

### 3.2. Clinical Data

**Figure 1:**
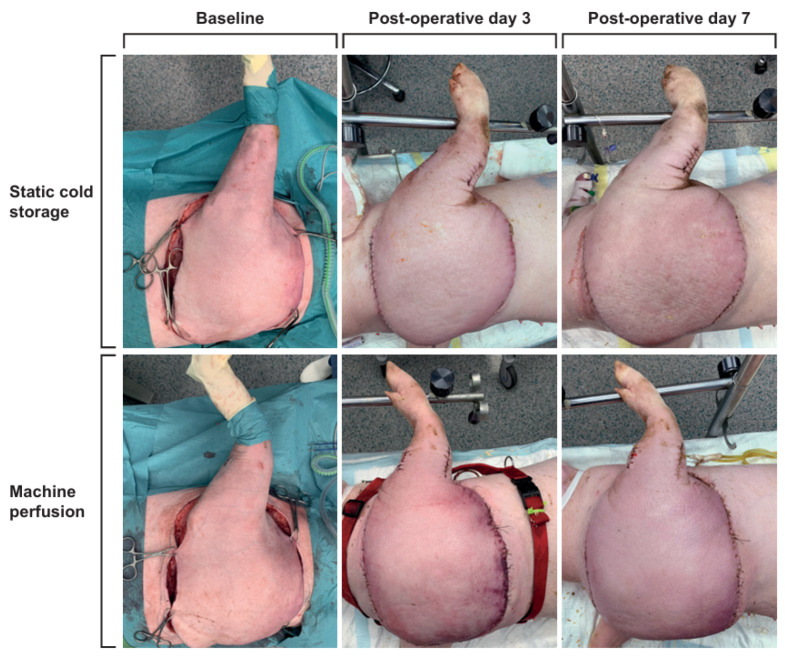
Color images capturing the perfused and static cold storage limbs at baseline and on post-operative days 3 and 7. One week later, assessments of limb viability (such as capillary refill times, temperature, and skin color) showed comparable results to those recorded at the baseline.

A total of twelve animals reached the end of the experiment, and four animals were excluded from testing on POD 7 due to various causes of unforeseen early death. In the SCS group, *n* = 1 due to vascular pedicle avulsion leading to death on POD 4, and *n* = 1 after a venous thrombosis on POD 5, which led to ending the experiment prematurely. In the MP group, *n* = 1 as a result of arterial thrombosis on POD 4, and *n* = 1 when the humane endpoint had been reached due to an arterial thrombosis in the hind limb after removing an inguinal arterial cannula on POD 3. Over the 7-day follow-up period, animals that underwent heterotopic replantation recovered well from the anesthesia, remaining hemodynamically and respiratorically stable. Evaluations of limb viability one week later, including capillary refill times, temperature, and skin color, closely matched the baseline measurements. Skin necrosis, ranging from 1–5%, was observed in three limbs (SCS *n* = 1; MP *n* = 2) on POD 7. Throughout the follow-up, ICG confirmed microcirculation in all limbs, indicating no signs of occlusions in veins or arteries (Figure 1).

### 3.3. Histopathology

**Figure 2:**
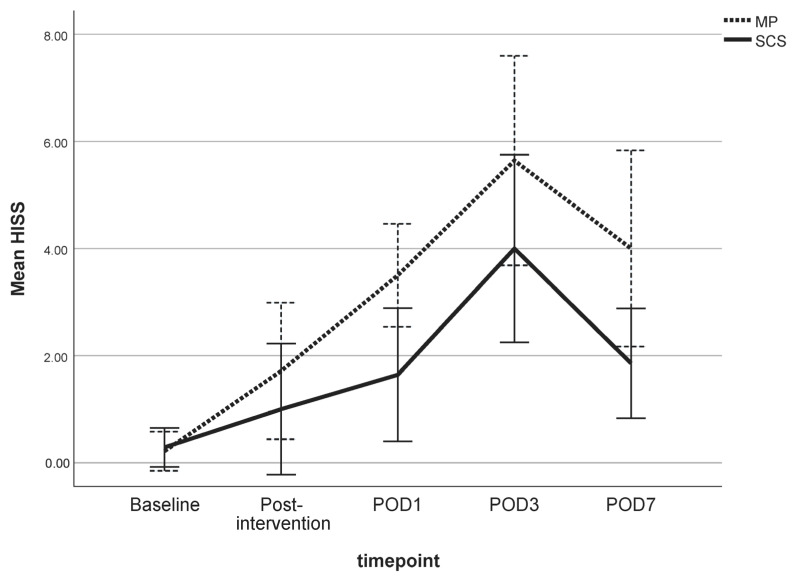
Mean Histology Injury Severity Scores (HISS) of cross sections of skeletal muscle biopsies preserved via static cold storage (SCS) or machine perfusion (MP) and stained with H&E. The values are shown for the baseline (MP *n* = 8; SCS *n* = 8), post-intervention (MP *n* = 8; SCS *n* = 8), and post-operative day (POD) 1 (MP *n* = 8; SCS *n* = 8), POD 3 (MP *n* = 8; SCS *n* = 8), and POD 7 (MP *n* = 6; SCS *n* = 6) timepoints. A 95% confidence interval is displayed. A Mann–Whitney U test was performed to determine the significance of changes between groups.

**Figure 3:**
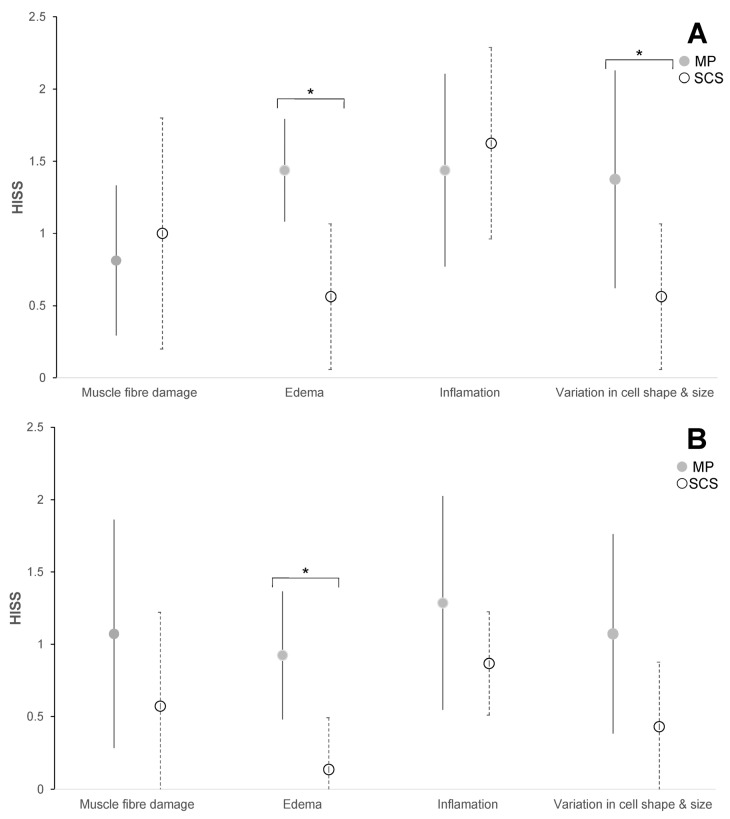
Mean sub Histology Injury Severity Scores (HISS) of cross sections of skeletal muscle biopsies preserved with static cold storage (SCS) or machine perfusion (MP) and stained with H&E on post-operative days (PODs) 3 and 7. (**A**) Subscores on POD 3 (MP *n* = 8; SCS *n* = 8): muscle fiber damage (*p* = 0.631), edema (*p* = 0.003), inflammation (*p* = 0.820), and variation in cell shape and size (*p* = 0.036). (**B**) Subscores on POD 7 (MP *n* = 6; SCS *n* = 6): muscle fiber damage (*p* = 0.347), edema (*p* = 0.001), inflammation (*p* = 0.319), and variation in cell shape and size (*p* = 0.091). Asterisks (*) indicate significance. A Mann–Whitney U test was performed to determine the significance of changes between groups.

**Figure 4:**
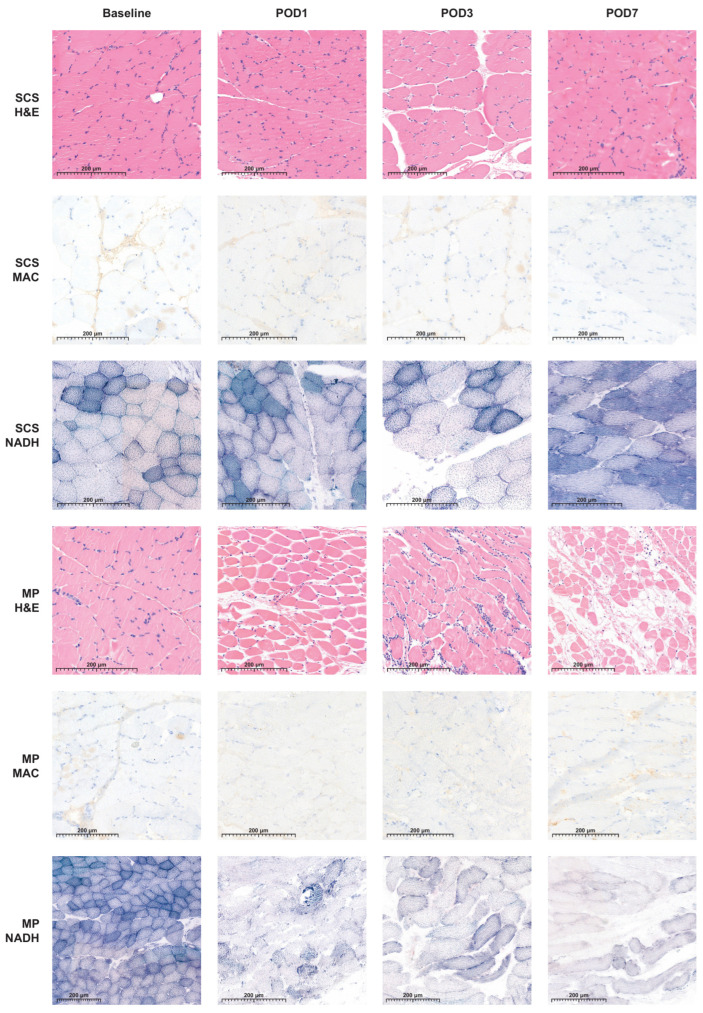
Representative H&E, MAC, and NADH images of skeletal muscle biopsies, preserved with static cold storage (SCS) or machine perfusion (MP), at baseline and on post-operative days 1, 3, and 7.

The HISS of both preservation groups varied over the 7-day follow-up and were statistically significantly different compared to the baseline measurements (*p* < 0.05) (Figure 2). Muscle biopsy specimens preserved by MP had a mean HISS of 4.9 (SD 2.8), and specimens preserved by SCS had a mean HISS of 3.5 (SD 2.3) (*p* = 0.029) on POD 3. The HISS subscores for the MP group are statistically significantly higher compared to those for the SCS group for edema (*p* = 0.003) and for the variation in cell shape and size (*p* = 0.036) on POD 3 (Figure 3A). The mean that the HISS decreased to 3.5 (SD 2.3) in the MP group and to 1.6 (SD 1.2) in the SCS group (*p* = 0.038) on POD 7. HISS subscores on POD 7 (Figure 3B) showed statistically significant edema (*p* = 0.001) in the MP group compared to that in the SCS group (Figure 4).

**Figure 5:**
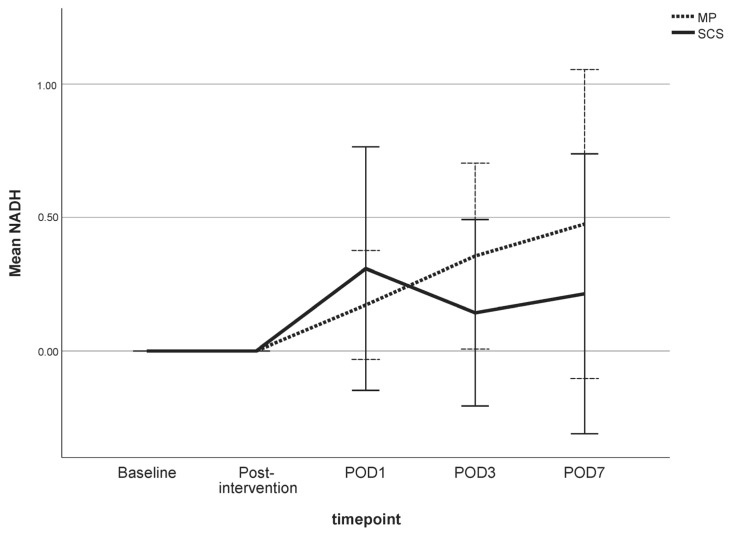
Mean NADH scores of cross sections of skeletal muscle biopsies preserved with static cold storage (SCS) or machine perfusion (MP) and stained with NADH. The values are shown for the baseline (MP *n* = 8; SCS *n* = 8), post-intervention (MP *n* = 8; SCS *n* = 8), and on post-operative day (POD) 1 (MP *n* = 8; SCS *n* = 8), POD 3 (MP *n* = 8; SCS *n* = 8), and POD 7 (MP *n* = 6; SCS *n* = 6) timepoints. A 95% confidence interval is displayed. Only descriptive analysis was performed to present changes between groups.

The NADH scores of both preservation groups varied over the 7-day follow-up and were statistically insignificantly different from the baseline measurements (*p* > 0.05) (Figure 5). Muscle biopsy specimens preserved by MP had a mean NADH score of 0.4 (SD 0.4), and specimens preserved by SCS had a mean NADH score of 0.1 (SD 0.4) (*p* = 0.318) on POD 3. On POD 7, the mean NADH score increased slightly to 0.5 (SD 0.6) in the MP group and to 0.2 (SD 0.6) in the SCS group (*p* = 0.456). No cytoarchitectural alterations were observed at baseline, post-intervention, or on POD 1 in either experimental group. Some individual variations emerged by POD 3, yet these alterations did not demonstrate statistically significant differences (Figure 4).

**Figure 6:**
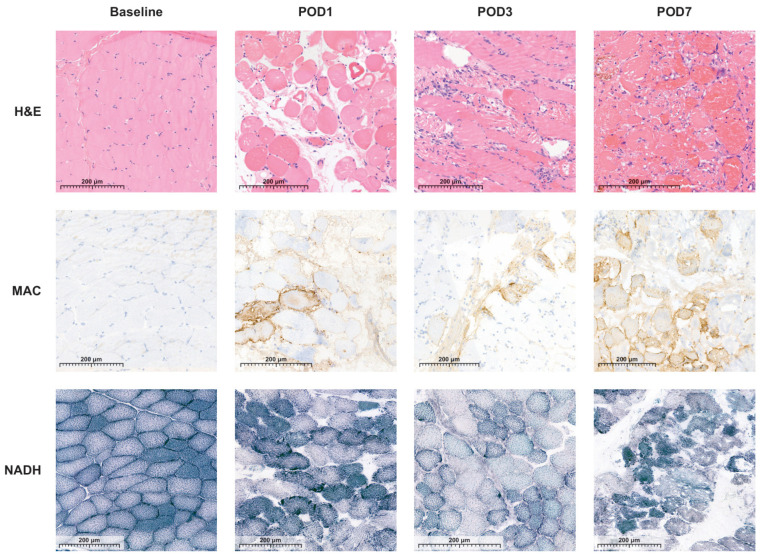
H&E, MAC, and NADH images of skeletal muscle biopsies preserved with static cold storage (SCS) at baseline and on post-operative days 1, 3, and 7.

Muscle biopsy specimens preserved with SCS or MP and stained with MAC consistently revealed no to minimal necrotic tissue across the different timepoints, resulting in predominantly negative MAC results for the majority of the specimens. Biopsy specimens obtained on POD 3 and 7 revealed no necrotic muscle fibers, with the exception of pigs number 6 (Figure 6) and 9. The presence of necrotic fibers in these two pigs corresponded with the observed muscle damage in the H&E and NADH-stained biopsies on POD 3. 

### 3.4. Blood Gas Analysis 

**Figure 7:**
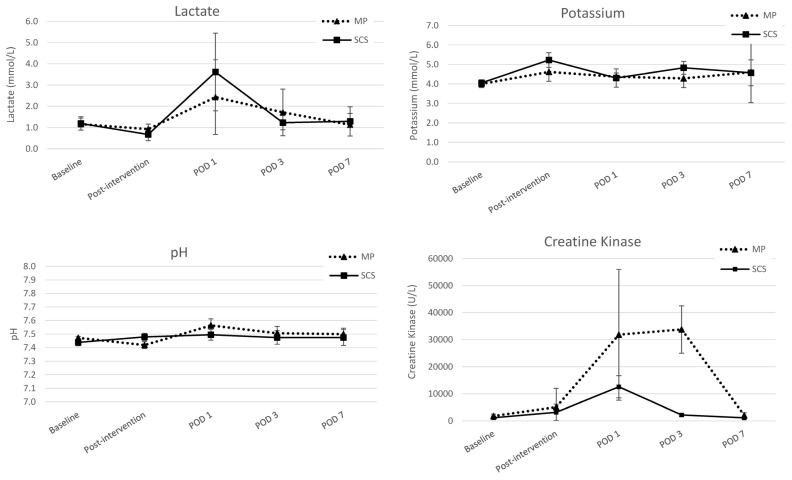
Biochemical parameters of blood of the limbs after preservation with static cold storage (SCS) or machine perfusion (MP) at baseline (MP *n* = 8; SCS *n* = 8), post-intervention (MP *n* = 8; SCS *n* = 8), post-operative day (POD) 1 (MP *n* = 8; SCS *n* = 8), POD 3 (MP *n* = 8; SCS *n* = 8), and POD 7 (MP *n* = 6; SCS *n* = 6). The mean per group is displayed with 95% confidence intervals. A Kruskal–Wallis test was performed to determine the significance of changes between groups.

Muscle damage markers exhibited a significant increase in the MP group during the initial 3 days following replantation when compared to the results for the SCS group. Specifically, CK levels were notably higher in the MP group at 33,781 mmol/L, as opposed to 2163 mmol/L in the SCS group (*p* < 0.001) (Figure 7). Lactate levels increased in both experimental groups on POD 1 (2.43 mmol/L versus 3.62 mmol/liter; *p* = 0.372). Meanwhile, pH levels remained stable at approximately 7.46 in the MP group but increased to 7.56 in the SCS group at 24 h after replantation.

## 4. Discussion

Identifying IRI in histological samples is an important task in VCAs, as the degree of muscle damage influences the vitality of the VCA and the transplant outcomes. This study found that NADH and MAC were able to visualize muscle damage in biopsy specimens with elevated HISS. However, they did not offer any additional information when assessing IRI compared with the results for H&E. MAC revealed positive muscle fibers in some cases, which corresponded to the morphology encountered in the H&E slides, hereby confirming the diagnosed alterations. Interestingly, MAC exhibited a contrasting pattern of results, as it did not indicate any muscle damage on POD 3 and 7 for pigs 1, 2, and 5, while the H&E stained biopsy specimens did show necrotic fibers. Sampling error seems to be the most likely explanation, as the alterations seem to be heterogenous within a tissue fragment, as also shown in the H&E sections.

Previous studies have examined IRI in porcine [5,6,8,9] and human [11] VCAs using H&E. Due to the absence of an internationally accepted standardized scoring system for evaluating muscle fibers, multiple additional immunohistochemical stainings have been utilized besides H&E to assess muscle damage. In a study from Kueckelhaus et al. [8], histological muscle damage was defined as vacuolization, hypercontractility, and necrosis of the muscle fibers. The assessment by Krezdorn et al. [6] used distinct histological markers defining signs of damaged muscle fibers as necrosis of the fibers and cells, hypercontractility, and vacuolization, whereas the HISS used by Kruit et al. [5] and in the present study also evaluates the presence and degree of interstitial edema, inflammation, and muscle fiber heterogeneity, in addition to the damage of muscle fibers. A study by Michel et al. [25] showed more edema in the MP group, while the cytological features were preserved. It is important to acknowledge that direct comparisons of the histological findings are challenging due to the differences in the scoring systems that were used. It is noteworthy that the scoring methods of both Krezdorn et al. [6] and Kruit et al. [5] indicated an increase in heterogeneity and interstitial edema, resulting in the decision to implement these factors into the HISS over time. 

NADH is a less frequently used staining method, yet it has demonstrated its utility for the assessment of oxidative histochemical activity and mitochondrial architecture in rat [18,26,27,28] and human [29] studies. As mitochondria play a role in the pathophysiology of IRI, NADH histochemistry analysis was performed in the present study to determine whether myocyte injury can be detected more accurately/in an earlier stage than is possible via conventional H&E. In ischemia–reperfusion studies using a lower-limb rat model, muscle damage was assessed through various methods. One approach involved categorizing the reactivity of NADH as intense, moderate, or weak [18], similar to the scoring method used in the present study. Another study assessed muscle viability through histochemical staining with nitroblue tetrazolium, quantified as a percentage using computerized planimetry [28]. Alternatively, muscle damage was assessed through morphometric evaluation of the NADH–tetrazolium reductase reaction by randomly photographing images of ten different fields and calculating the viability of the fibers as a proportion of the total area of positive NADH staining and the total area of muscle fibers in each picture [26,27]. Using morphometric software for calculating the degree of muscle damage could offer a more consistent and quantitative approach for assessing the muscle fibers than the objective assessment used in the present study. In the present study, the NADH outcomes did not always correspond to the HISS, which could be a possible consequence of sampling error or caused by the differences in the scoring systems that were used to assess the muscle damage for each staining.

A less commonly used, but potentially valuable, staining is MAC. As IRI activates complements the subsequent formation of C5b-9 channels (i.e., MAC) on the cell membranes, leading to myocyte necrosis, MAC staining proves to be a reliable diagnostic marker for detecting necrotic muscle fibers [30]. Several studies assessed muscle damage by evaluating the intensity and/or distribution of the MAC staining using a semi-quantitative scale [31,32,33,34,35]. The literature shows that MAC serves as a marker for early myocardial ischemia [34,35] and proves to be a valuable and specific post-mortem marker for myocardial infarction [33]; however, there is still a scarcity of research exploring the utilization of MAC in the context of VCAs. In the present study, MAC was evaluated using a self-developed scoring system by counting the number of positive fibers. Even though MAC showed the muscle damage in the biopsy specimens, it did not contribute to the assessment of IRI, except for affirming that the H&E stained biopsy specimen revealed (highly suspicious) necrotic fibers.

In the current study, biochemical markers such as potassium, creatinine kinase, and lactic acid levels were assessed during 24 h machine perfusion and after in vivo replantation. A linear increase in these markers was observed during perfusion, which did not correlate with muscle histology outcomes post-replantation in the perfusion group. Other studies, including those by Krezdorn et al. [6] and Kruit et al. [5], noted a similar discrepancy between biochemical markers and muscle histology. The consistent release of markers into the perfusate or blood from the severed limb end may obscure the detection of smaller amounts released within the limb itself, necessitating caution when interpreting these markers in relation to graft survival and histopathologic scoring. 

The implementation of the different staining methods requires the consideration of several factors. The complex techniques that are required to conduct the NADH and MAC staining methods must be performed by highly trained specialists, thus augmenting the temporal and financial resources devoted to the procedure. The costs of a single batch of 20 pieces for H&E, NADH, and MAC are each, respectively, approximately EUR 50, EUR 338, and EUR 590. This cost difference is notably increased by the disparate temporal demands of the staining methods, with H&E requiring 20 min, NADH requiring 4 h, and MAC extending over a 7 h period. The time required to use MAC may not align with the objective of obtaining a real-time impression of the muscle tissue of a VCA. These cost estimates pertain exclusively to the staining methods and do not include the expenses associated with the digitalization and subsequent assessment of the biopsies by a pathologist. Another important consideration is the storage conditions needed for the NADH- and MAC-stained biopsy specimens. These specimens will be snap-frozen and require storage at −80 °C, while the H&E stained biopsy specimens can be safely stored in formaldehyde at room temperature. With the aim of investigating IRI in VCAs involving transportation, the feasibility of storing muscle biopsy specimens at room temperature would offer greater convenience. Considering all these factors, it is important to acknowledge the extended requirements associated with the utilization of both NADH and MAC in contrast to the comparatively more efficient and cost-effective H&E staining.

This study is limited by its sample size and the lack of validated scoring systems to assess the histological outcomes. Firstly, varying scoring system ranges and limited data hinders direct result comparison. As a result, it was not possible to determine whether NADH or MAC stainings are effective for comparing groups or monitoring changes over time within the same group. Secondly, while HISS provides more information on the type of muscle damage, NADH and MAC scoring systems are focused on specific cell injuries. Notably, elevation in any of the HISS subscores impacts the overall HISS, affecting muscle damage interpretation. Thirdly, a lack of healthy limb biopsies as controls was deliberate to avoid animal weakening. Ethical constraints prevented using 24 h SCS for comparative purposes with 24 h MP. Finally, the correlation between the different histological staining methods remains uncertain, and there were no additional clues found in this study using NADH and/or MAC to support signs of IRI beyond those observable with H&E. Comparing outcomes across studies is ultimately challenging due to variations in graft models, ischemic durations, perfusate solutions, and perfusion parameters, potentially influencing outcomes.

The present study documents using H&E in combination with the simplified HISS, using representative histological images to assess muscle damage. An added diagnostic value of NADH and MAC in detecting IRI in porcine limbs in comparison to H&E could not be demonstrated. While H&E has its limitations, such as its inability to reveal deviations in myofibrillar architecture and intracellular muscinds, along with its challenge in differentiating between types of myocyte injury, the findings of this study suggest that future research in this area should continue to utilize H&E alongside the HISS for assessing IRI in both porcine and human VCAs. H&E stands out as the most efficient and cost-effective staining method when compared to NADH and MAC. At present, there are no superior alternatives to H&E. Thus, on a histopathological level, H&E remains the preferred staining method. However, until a better method is discovered, it is imperative to interpret HISS values in conjunction with other results, such as clinical and biochemical data. Expanding the scope of research in this field can be achieved by extending the duration of post-transplantation follow-up to enable a more focused examination of the regenerative capabilities of muscle tissue. In the context of investigating IRI in porcine muscle tissue within a whole-limb transplantation model, H&E remains the staining of choice.

## 5. Conclusions

This study has thoroughly documented the simplified HISS and its application in the machine perfusion of VCA. The use of NADH and MAC did not provide any additional diagnostic value to HISS in detecting IRI in porcine limbs. Applying the HISS, as proposed in Table 1, appears to be an effective method for assessing IRI in muscle fibers. Muscle tissue preserved by either 24 h MP or 4 h SCS showed no to minimal damage after a 7-day follow up.

## Figures and Tables

**Table 1:** Histology Injury Severity Score (HISS) for hypoxia-induced muscle injury.

**Interstitial edema**
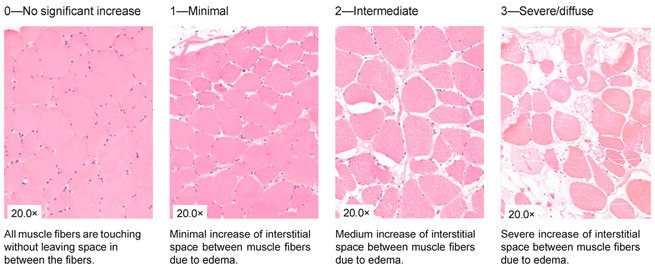
**Inflammation**
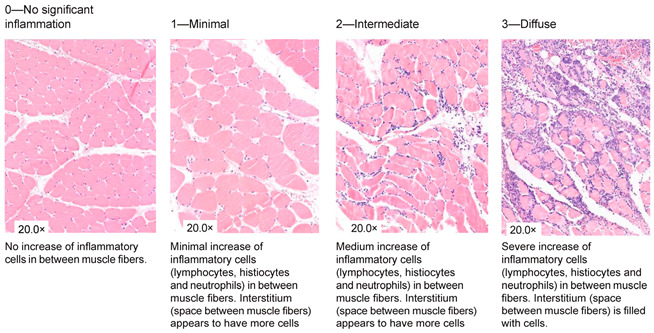
**Inflammation**
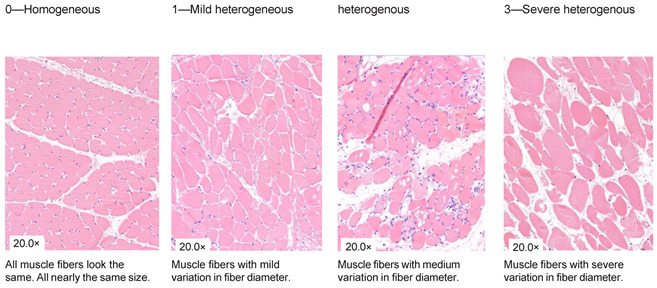
**Damaged muscle fibers**
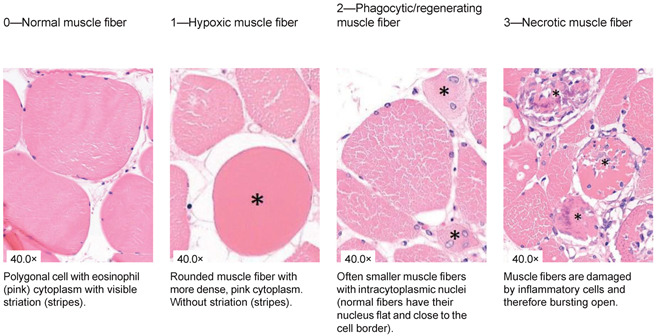

You count the number of cells in 10 High Power Fields with a 20× magnification and score as stated below: 0.—0–5 myocytes/10 HPF (20× magnification); 1.—6–20 myocytes/10 HPF (20× magnification); 2.—21–50 myocytes/10 HPF (20× magnification); 3.—51 myocytes/10 HPF (20× magnification). Note: Muscles can be cut in 2 ways; cross-sections or longitudinal-sections. Cross-sections are the most reliable, but this is not always possible when working with biopsies. The scoring method therefore stays the same. You can only count with 10 HPF in a large piece of tissue. This is not always possible when working with biopsies, therefore the biopsy is scored as a whole. You score the all-over impression of edema, inflammation a variety in fiber diameter. You also count all damaged muscle fibers in total. * indicates damaged muscle fibers.

**Table 2:** NADH scoring system for evaluating myofibrillar architecture and staining intensity in muscle fibers.

Unclassifiable	0—Normal NADH Staining (<5 Abnormal Fibers)	1—Minimal Alterations (5–14 Abnormal Fibers)	2—Intermediate Alterations (15–49 Abnormal Fibers)	3—Severe/Diffuse Alterations (>50 Abnormal Fibers)
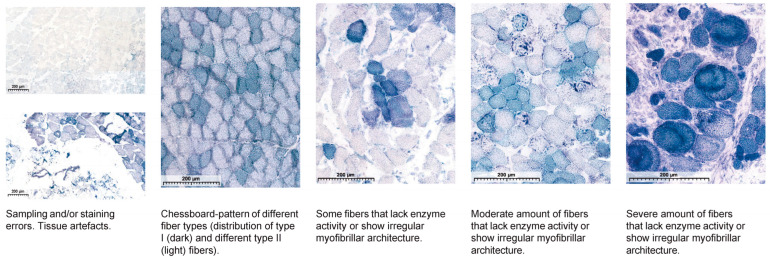				

Histological–intensity of the staining and staining pattern. Scoring is based on: Normal myofibrillar architecture; Irregular myofibrillar architecture (disarray) with white centered fibers and granular aggregates (NADH positive granules); Intensity: “It was previously demonstrated that the intensity of NADH-TR staining on frozen sections decreases after a relatively long period of ischemia, even without reperfusion/”.

**Table 3:** MAC scoring system for necrotic fibers.

Negative	Positive
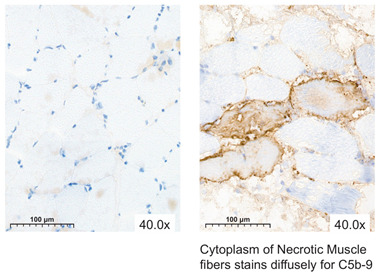	

**Table 4:** Baseline characteristics of the static cold storage group, the machine perfusion group, and their corresponding *p*-values [24]. Abbreviations: static cold storage (SCS); machine perfusion (MP); warm ischemia time (WIT). A Mann–Whitney U test was performed to determine significance of the changes between groups.

	SCS (*n* = 8)	MP (*n* = 8)	*p*-Value
Characteristics	Mean (SD)	Mean (SD)	
Harvest (min)	92 (17)	87 (14)	0.636
Ex vivo storage time (h)	4 (0)	24 (0)	<0.001
WIT before storage (min)	2.6 (1.1)	12.9 (7.4)	0.005
WIT before reperfusion (min)	52 (27)	58 (16)	0.172
Limb weight before intervention (g)	2580 (285)	2554 (395)	0.916
Limb weight after intervention (g)	2542 (274)	3676 (312)	<0.001
Weight difference (%)	−2%	44%	<0.001
Temperature before intervention (°C)	35.4 (1.0)	34.6 (1.1)	0.171
Temperature after intervention (°C)	6.5 (1.5)	12.0 (1.4)	<0.001

## Data Availability

All data obtained during this study at the Radboud University Medical Center have been stored and are available at the Digital Research Environment (DRE, https://mydre.org/ (accessed on 31 May 2024)). This is a cloud-based, globally available research environment where data can be safely collected and stored. The files are indexed per research project on the virtual machine “Extracorporal Thrombolysis”, owned by the Radboud University Medical Center. The raw data obtained for this study will be saved for 15 years after publication (until 2038). The datasets analyzed during the experiments in this study are available upon reasonable request.
